# The Dynamical Mechanism of Auto-Inhibition of AMP-Activated Protein Kinase

**DOI:** 10.1371/journal.pcbi.1002082

**Published:** 2011-07-21

**Authors:** Cheng Peng, Teresa Head-Gordon

**Affiliations:** 1 MOE-Microsoft Key Laboratory for Intelligent Computing and Intelligent Systems, Department of Computer Science and Engineering, Shanghai Jiao Tong University, Shanghai, China; 2 Department of Bioengineering, University of California, Berkeley, Berkeley, California, United States of America; National Cancer Institute, United States of America and Tel Aviv University, Israel

## Abstract

We use a novel normal mode analysis of an elastic network model drawn from configurations generated during microsecond all-atom molecular dynamics simulations to analyze the mechanism of auto-inhibition of AMP-activated protein kinase (AMPK). A recent X-ray and mutagenesis experiment (Chen, et al *Nature* **2009**, *459*, 1146) of the AMPK homolog *S. Pombe* sucrose non-fermenting 1 (SNF1) has proposed a new conformational switch model involving the movement of the kinase domain (KD) between an inactive unphosphorylated open state and an active or semi-active phosphorylated closed state, mediated by the autoinhibitory domain (AID), and a similar mutagenesis study showed that rat AMPK has the same auto-inhibition mechanism. However, there is no direct dynamical evidence to support this model and it is not clear whether other functionally important local structural components are equally inhibited. By using the same SNF1 KD-AID fragment as that used in experiment, we show that AID inhibits the catalytic function by restraining the KD into an unproductive open conformation, thereby limiting local structural rearrangements, while mutations that disrupt the interactions between the KD and AID allow for both the local structural rearrangement and global interlobe conformational transition. Our calculations further show that the AID also greatly impacts the structuring and mobility of the activation loop.

## Author Summary

AMP-activated protein kinase (AMPK) maintains the balance between ATP production and energy consumption in eukaryotic cells by responding to the rise of intracellular AMP. We report on a novel method that uses normal mode analysis of an elastic network model drawn from microsecond all-atom molecular dynamics simulations to analyze the activation mechanism of the AMPK homolog SNF1, which is believed to have the same mechanism as mammalian AMPK. There has been important new X-ray crystallographic and mutagenesis information on the self-regulation of AMPK based on its auto-inhibitory domain, although that view is primarily static. We provide a dynamical analysis to show that AID inhibits catalytic function by restraining KD into an unproductive open conformation and limiting functional local structural rearrangement, and that mutations that disrupt the interactions between the KD and AID free the KD to undergo both the global interlobe conformational transition and functional local structural rearrangement. This suggests new ways in which drugs might be used to regulate this important molecular machine.

## Introduction

AMP-activated protein kinase (AMPK) is a highly conserved enzyme in eukaryotic cells that regulates cellular and whole-body energy homeostasis by phosphorylating a wide variety of substrates [1], [2], [3], [4]. The homolog of AMPK in yeast [5], [6], sucrose non-fermenting 1 (SNF1), has been widely used as a model system for mammalian AMPK due to their large similarity in both structure and function, in which the enzyme homologues are heterotrimers [7], [8], [9] consisting of a catalytic subunit (α-subunit) and two regulatory subunits (β- and γ- subunits). The catalytic α-subunit has a kinase domain (KD) that includes both N-terminal and C-terminal lobes that phosphorylates downstream substrates, an autoinhibitory domain (AID) that inhibits KD catalysis, and a regulatory domain that communicates with the β and γ subunits. The β-subunit acts as a scaffold to influence how the AMPK complex assembles, and contains a central glycogen-binding domain and a C-terminal domain interacting with both the α and γ subunits. The γ subunit is composed of a N-terminal domain, a short segment binding to the β subunit, and two Bateman domains that bind AMP or ATP. The binding of AMP to the Bateman domains can allosterically activate the catalytic function in the α-subunit, and instigates the phosphorylation of downstream proteins to mediate other biological pathways. This signaling progression also requires the phosphorylation of a threonine residue in the activation loop of KD by an upstream kinase. In mammals, each of the subunits has multiple isoforms (α1, α2, β1, β2, γ1, γ2 and γ3) [6], so there may be as many as 12 combinations, each with a different function.

**Figure 1:**
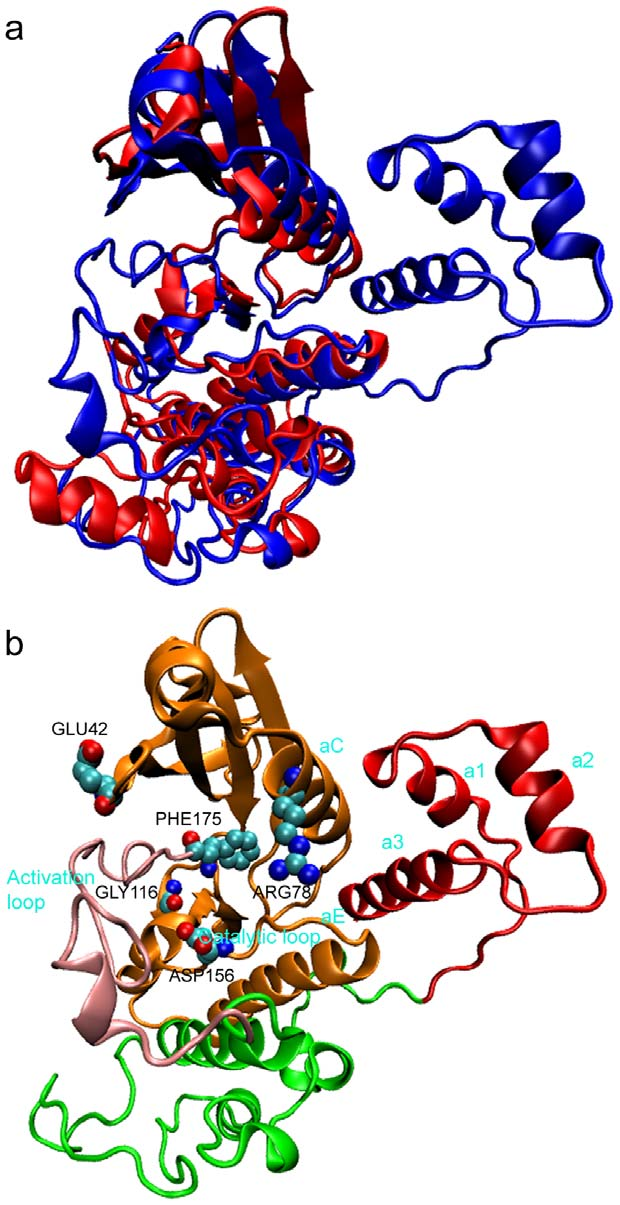
Ribbon structures of the KD-AID fragment. (a) Comparison of the unphosphorylated KD-AID fragment (PDB Id: 3H4J, blue ribbons) that represents the open state with the homologous phosphorylated KD fragment (PDB Id: 3DAE, red ribbons) representing the closed state. (b) The N-terminal lobe (residues 27–114) and part of the C-terminal lobe (residues 115–175) of the KD domain are shown in orange, and are used to measure the interlobe conformational transition. The activation loop (residues 176–206) is shown in pink, with the rest of KD shown in green, and the AID fragment shown in red.

Biochemical experiments have shown that the isolated full-length α-subunit and even the α1 isoform (residues 1–392) have little activity due to the presence of the conserved AID domain [10], [11]. Recently, an exciting X-ray crystallography study [12] has successfully crystallized an unphosphorylated fragment containing both KD and AID from *Schizosaccharomyces pombe* (PDB Id: 3H4J), and a phosphorylated fragment containing only KD from *Saccharomyces cerevisiae* (PDB Id: 3DAE), providing a static structural view of how AID inhibits the conformational transition of the N-terminal and C-terminal lobes in the KD domain to the functional closed state (Figure 1a) [13]. Mutagenesis of key residues of AID were found to restore catalytic function of the KD fragment, thereby isolating key residue interactions between these two domains. The same interface point mutations of the rat AMPK α1 subunit show exactly the same catalytic trends as the *S.Pombe* KD-AID fragment, which was further confirmed in the rat AMPK holoenzyme in that these same mutations both increase the catalytic activity and slow the dephosphorylation of the α-subunit, independent of AMP concentration. Based on the X-ray crystal structures and catalytic activity upon mutagenesis, the authors proposed a new conformational switch model for the regulatory mechanism of AMPK activity in which the interaction of AID with KD requires the latter to adopt a relatively open conformational form that is inactive. The eventual binding of AMP to the γ-subunit changes the interactions between the AID and KD, at present by an unknown molecular mechanism, to remove the inhibitory effect of AID to allow the interlobe conformational transition to the closed state.

However, there is no direct dynamical evidence to support the conformational switch model. Structural studies of the protein kinase family [14] have shown that the catalytic activity involves the functional rearrangement (Figure 1b), of local structures such as the Gly-rich loop, the helix αC, the catalytic loop, the DFG-motif and the activation loop. Even if this model holds, it is not yet known whether AID inhibits catalytic function by inhibiting the interlobe conformational transition or rearrangements of these local structural elements, or both. In this work we address these questions by studying the interlobe conformational transition and corresponding rearrangements of the local structural elements in the wild-type KD-AID, and with AID mutated at key residues, or when AID is eliminated altogether, using a novel sampling approach [15] to study large protein conformational changes at an atomistic scale. The unphosphorylated KD-AID fragment from *S.Pombe* (PDB Id: 3H4J) is used as a model of the inactive-open state because of its open interlobe conformation, while the phosphorylated KD fragment from *S.Cerevisiae* (PDB Id: 3DAE) is used as the active-closed state reference, in accord with the experimental structural and mutagenesis analysis. While the reference structure 3DAE is still not completely active due to its disordered activation loop, experiments [12] have shown that this KD fragment has high catalytic ability, making it a reasonable model for the active-closed state. We find that AID hinders the interlobe conformational change in the KD, while mutations to key residues that disrupt the interactions between the AID and KD do in fact permit functional interlobe conformational transition between the KD open and closed states. Furthermore, we find that other functionally important local regions, including the Gly-rich loop, the helix αC, the catalytic loop, and the DFG-motif, show greater conformational flexibility or transition to an active-closed state under the same set of functional mutations. Interestingly, our calculations also show that the activation loop is greatly influenced by the presence of the auto-inhibitory domain, and that function-inducing mutations to AID impact the structure and mobility of the activation loop.

## Results

According to mutagenesis analysis [12], the wild-type KD-AID fragment and mutation N345A of the auto-inhibitory domain (mutant 1) showed little catalytic activity. By contrast, mutations of AID at positions L341D (mutant 2) and M316E (mutant 3), or elimination of AID altogether, exhibited strong catalytic ability. We use our instantaneous normal modes (INM) method [15] to project structural snapshots drawn from a molecular dynamics trajectory against the inactive-open state crystal structure 3H4J and the active-closed state reference structure 3DAE (see Methods). The two reference structures that measure the open and closed states exhibit an INM similarity value of ∼0.8 (and a root mean square deviation (RMSD) of 2 Å), indicating that INM values of 0.8 are dissimilar for this enzyme system.

**Figure 2:**
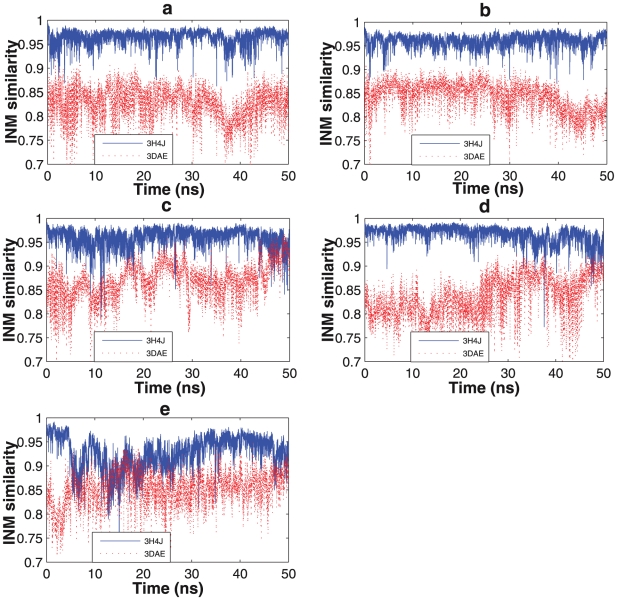
Instantaneous normal mode similarities calculated from a 50 ns molecular dynamics simulation. Solid blue line represents the structural similarity to the open state (3H4J) and the dashed red line is for the closed state (3DAE). (a)Wild-type KD-AID fragment, (b) Mutant 1, (c) Mutant 2, (d) Mutant 3, (e) KD fragment.

**Figure 3:**
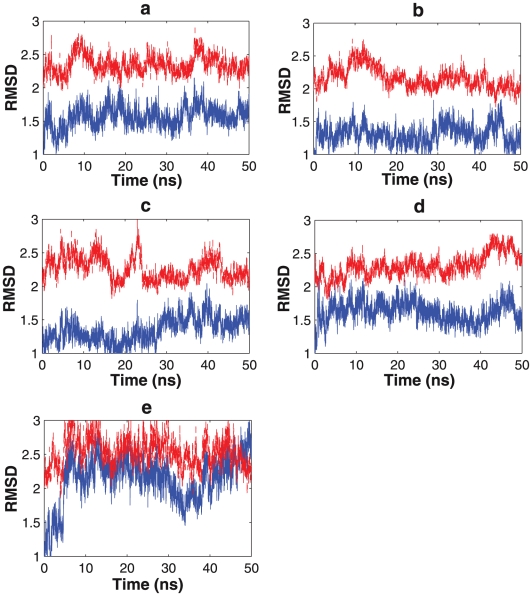
Root mean square deviation calculated from a 50 ns molecular dynamics simulation. Solid blue line represents the structural similarity to the open state (3H4J) and the dashed red line is for the closed state (3DAE). (a)Wild-type KD-AID fragment, (b) Mutant 1, (c) Mutant 2, (d) Mutant 3, (e) KD fragment.

Figure 2 illustrates the conformational changes of the initial trajectories starting from the open state 3H4J structure, as measured by the INM similarity metric. The wild-type KD-AID fragment and mutant 1 (Figures 2a and 2b) show very stable INMs, with high similarity (above 0.95) to the open state and a relatively low similarity (between 0.80 and 0.85) to the closed state, clearly indicating that there is no interlobe conformational transition to an active-closed state in each case. By contrast, the INM similarities to the open and closed reference states for mutant 2, mutant 3, and the isolated KD fragment (Figures 2c, 2d and 2e) show much greater flexibility, with mutants 2 and 3 showing a possible transition to the closed state over the initial 50 ns trajectory. This is to be compared to the RMSD measure over the same trajectory, which only shows a transition for the isolated KD fragment (Figure 3), indicating that the INM metric is more sensitive to relevant protein dynamical motions.

**Figure 4:**
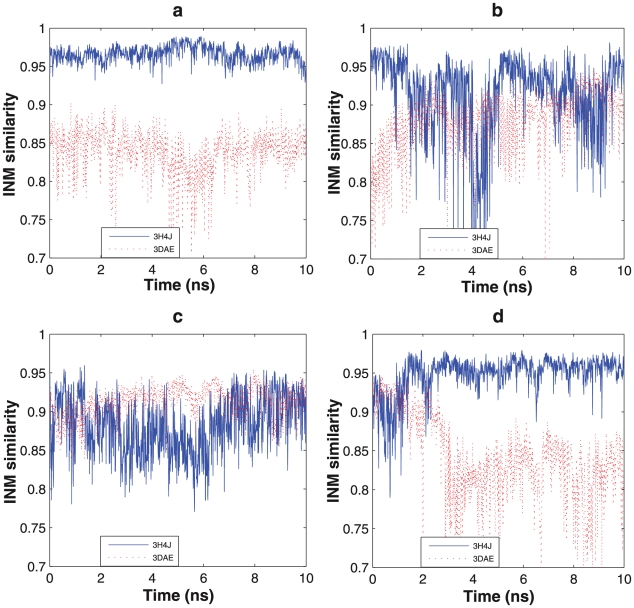
Instantaneous normal mode similarities calculated from many 10 ns molecular dynamics simulation showing signatures of (a) the open state, (b) transition from the open to closed state, (c) the closed state, and (d) transition from the closed to open state.

Therefore, to further investigate the interlobe conformational transition, we launched 90 independent 10 ns simulations starting from the open state for wild-type KD-AID, mutant 1, mutant 2 and mutant 3, and 70 independent 10 ns simulations starting from the open state for the isolated KD fragment. For the wild-type KD-AID fragment and mutant 1, all 90 of the 10 ns trajectories stay close to the open state with no fluctuations consistent with the closed state (Figure 4a). Mutants 2 and 3 and the KD fragment all show a fraction of trajectories that transition from the open to the closed state (Figure 4b). We then performed another 70 independent 10 ns simulation trajectories, this time from the newly obtained closed state for each of mutant 2, mutant 3 and the KD fragment. While most trajectories still remain in closed state (Figure 4c), a fair fraction of them revert back to the open state (Figure 4d). The detailed transition number in each case is reported in Table 1.

**Table 1:** Percentages of conformational transitions between open and closed states for KD-AID and Mutants.

Protein	Start State	Total # Trajectories	No transition	Transition
*KD-AID*	Open	90	100%	0%
*KD-AID Mutant 1*	Open	90	100%	0%
*KD-AID Mutant 2*	Open	90	92%	8%
*KD-AID Mutant 2*	Closed	70	83%	17%
*KD-AID Mutant 3*	Open	90	97%	3%
*KD-AID Mutant 3*	Closed	70	70%	30%
*KD fragment*	Open	70	80%	20%
*KD fragment*	Closed	70	91%	9%

Rows represent the different start states, open (3H4J) or closed (3DAE), for each protein (if accessible), the total number of 10 ns simulations starting from that configuration, and the percentage of observed outcomes that classify the final structure as open or closed.

**Figure 5:**
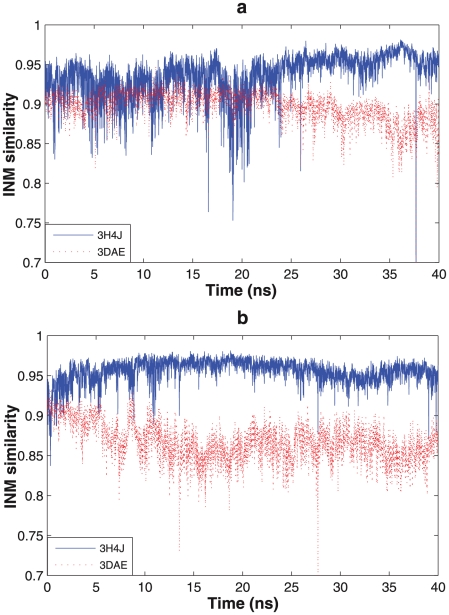
Trajectories started from the closed state for wild-type KD-AID and mutant 1 after back mutation from mutant2. (a) Wild-type KD-AID and (b) Mutant 1.

While we didn't observe any transition to the closed state in both wild-type KD-AID and mutant 1, we wanted to further clarify whether these two sequences either have a strong thermodynamic preference for the open state or whether the closed state is also preferred but is inaccessible due to a large free energy barrier from the open state that is unattainable on molecular simulation timescales. We therefore picked closed conformations from the stable mutant 2 trajectory as the start state for mutations of the functional mutants back to wild-type KD-AID (D341L) and mutant 1 (D341L and N345A), and simulated them for an additional 40 ns initial simulations. We find that both sequences revert back to the open state within 30 ns (Figure 5), showing that interactions with AID stabilizes the kinase domain in the inactive-open state.

**Figure 6:**
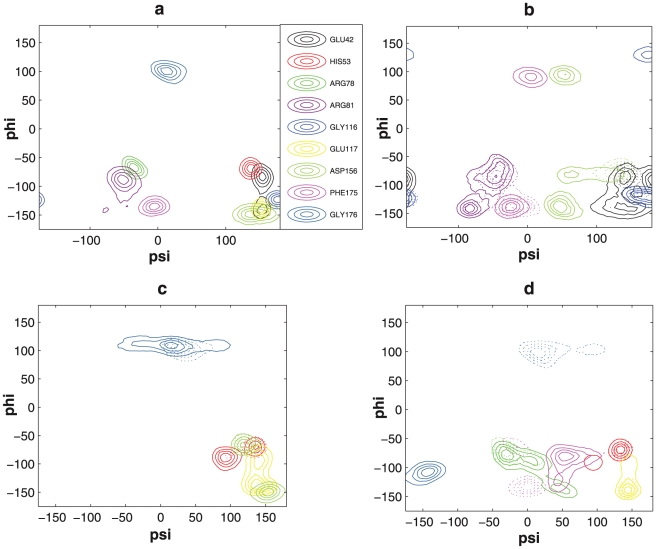
Contour map of statistics on dihedral angles of (a) open state in the wild-type KD-AID fragment and mutant 1, (b) open and closed states in the KD fragment, (c) open and closed states in mutant 2 and (d) open and closed states in mutant 3. Different from (a), dotted line represents open state and solid line denotes closed state in (b)–(d) to better illustrate the results. The specific color corresponds to the referred residue.

To investigate whether rearrangement of local structure is occurring along with the global interlobe conformational transition, we evaluated statistics on sampled backbone dihedral angle values for all sequences in the open state, and closed state if it exists for that sequence (Figure 6). Not surprisingly, there are no large backbone structural rearrangements when the interlobe conformation is open, regardless of sequence. However, residues associated with the Gly-rich loop (Glu42), the helix αC (Arg81), the catalytic loop (Asp156), the DFG-motif (Phe175), and the hinges (Gly115 and Gly116) connecting the two lobes, show greater structural flexibility when the interlobe conformation is closed, and sometimes shows evidence of a transition to an active or nearly active state when compared to the 3DAE reference structure.

Figure 6b shows that residues Glu42, Arg81, Asp156 and Phe175 of the isolated KD fragment have greater conformational flexibility in the closed state, with the hinge residue Gly116 exhibiting two conformational basins in the closed state, and Gly115 already in an active-closed state conformation even when the interlobe conformation is open (see Figure 1 in Text S1). Quite similar trends to the isolated KD fragment are observed for mutants 2 and 3 (Figures 6c and 6d, and Figures 2 and 3 in Text S1), although differences with the isolated KD do exist for the mutant 2 and mutant 3 sequences. In particular, there is almost no obvious dihedral angle change in the Gly-rich loop of mutants 2 and 3. In addition, mutant 2 shows greater flexibility of the two hinge segments, His53-Lys55 and Arg90-His91, with a stable backbone conformation between these two hinge segments that includes helix αC, while mutant 3 exhibits flexibility of helix αC but with stable nearby hinge residues (see Table 1 in Text S1). Furthermore, the catalytic loop residues in mutant 2 are highly flexible or even transition to a more active state while the backbone changes of the DFG-motif are relatively low. By contrast, large structural flexibility and rearrangement of the DFG-motif are observed in mutant 3, but without any observation of backbone rearrangement of the catalytic loop residues (see Table 1 in Text S1). Interestingly, it is not always the functional residues that are more mobile: in mutant 2, Asp156, Leu157 and Lys158 appear to transition to an alternative conformation during the interlobe conformational transition from open to closed state, unlike the isolated KD fragment (see Table 1 in Text S1).

**Figure 7:**
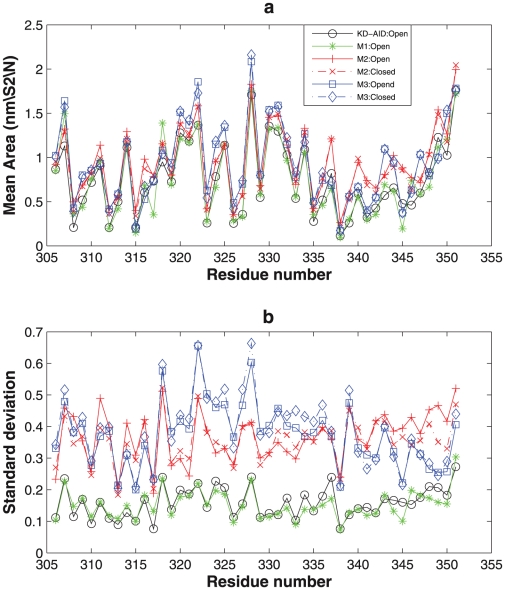
Solvent accessible surface area for different cases calculated from 700 ns trajectories each. (a) Average values. (b) Standard deviations.

It appeared from structural snapshot observations along the trajectory that the solvent exposure at the interface between the KD and AID changed between the functional and non-functional sequences. Figure 7 shows the average solvent accessible surface area (SASA) and corresponding standard deviation [16] for the AID (residues: 306 to 351) for the WT KD-AID and three mutants. Overall the WT KD-AID and mutant 1 (the non-functional cases) have much reduced solvent exposure of the AID, especially in the region of the last helix (residues: 335 to 350). In contrast, mutants 2 and mutant 3, regardless whether they are in the open or closed states, have much higher SASA values. It is clear that the functional mutations result in loss of direct association of the AID with the KD fragment, thereby freeing the N-terminal and C-terminal lobes to execute the functional interlobe transition and rearrangements of the local structural elements of KD.

Finally we considered whether there is any evidence of increased mobility of any particular region of KD or AID in the closed vs. open states. Table 2 reports the backbone configurational entropy per atom in the different regions of the KD-AID structure for the wild-type and three mutants; we do not include the KD fragment since it is so flexible without the auto-inhibitory domain that it makes comparison to the other KD-AID sequences problematic. It can be seen that the backbone entropy is largely the same among all sequences in either the open or closed state, except for large differences seen in the activation loop region. The nonfunctional sequences (wild-type KD-AID and mutant 1) show much greater backbone entropy in the activation loop than the functional sequences (mutant 2 and mutant 3) in the open state, while for mutants 2 and mutant 3 the activation loop has higher backbone entropy in the closed state relative to the open state. Since we did not observe transition to the closed state for the wild-type KD-AID and mutant 1, there is no direct comparison of backbone entropy between the closed states for the nonfunctional and functional sequences. It appears that there is significant activation loop mobility changes for the functional mutants as they undergo transitions between the open and closed conformations.

**Table 2:** Configurational entropy of backbone atoms (N, C and Ca) for the KD-AID and mutants.

Protein	Cleft (27–175)	Activ. Loop (176–206)	Remain KD (210–297)	KD (27–297)	AID (298–325)	All (27–325)
*KD-AID*	20.71 (0.21)	29.07 (0.21)	21.13 (0.17)	21.07 (0.09)	23.27 (0.17)	20.97 (0.09)
*Mutant 1 open*	19.30 (0.08)	26.87 (0.84)	21.53 (0.09)	20.23 (0.12)	22.37 (0.34)	20.17 (0.05)
*Mutant 2 open*	20.07 (0.17)	25.63 (0.40)	21.33 (0.05)	20.4 (0.08)	23.30 (0.65)	20.40 (0.08)
*Mutant 3 open*	19.93 (0.12)	24.20 (0.36)	21.53 (0.31)	20.33 (0.17)	21.60 (0.14)	20.13 (0.17)
*Mutant 2 closed*	20.23 (0.25)	28.10 (0.78)	21.40 (0.22)	20.70 (0.22)	22.43 (0.33)	20.50 (0.22)
*Mutant 3 closed*	20.03 (0.19)	27.27 (0.94)	21.27 (0.21)	20.33 (0.46)	21.30 (0.16)	19.97 (0.56)

The calculation is based on trajectories of 700 ns each and is in units of kJ/mole/K per atom.

## Discussion

Our theoretical study has shown that the auto-inhibitory domain does play an important role in regulating both the interlobe conformational transition and the functional rearrangements of local structural elements to inhibit the catalytic function of AMP-activated protein kinase. As found by previous experiments [12], disruption of several key interactions in the AID that are manifested as mutant 2 and mutant 3, or elimination of AID altogether, do show better catalytic activity. Our dynamical analysis confirms that the wild-type KD-AID fragment and mutant 1 do not undergo the functional global interlobe conformational transition and local structural rearrangements because the open state of the KD is highly preferred relative to the catalytic closed conformation. In fact, the KD fragment appears to prefer the closed state since the transition rate from the closed to open state is much lower than the reverse rate (see Table 1), highly consistent with the experimental result [17] which showed that the KD can refold as a means of regulating the AMPK autoinhibitory mechanism. Finally, the back mutation of functional mutant 2 or mutant 3 to the wild-type KD-AID and mutant 1 sequences destroy its ability to undergo a conformational transition by stabilizing the inactive-open state.

The SASA calculation shows that the functionalizing mutations of AID make it more solvent exposed, suggesting that the increase in the interlobe conformational transition, structural rearrangements of various local regions of KD, and the corresponding catalytic ability come from the disruption of interdomain interactions between the KD and AID. The loss of inter-domain interactions in turn affects the activation loop, which undergoes significant dynamical changes when executing the conformational transition as measured by the significant increase in backbone entropy in the closed state relative to the open state, supported by larger variation of backbone dihedral angle values. However, the mobility of residues in the activation loop differed greatly among different sequences, so that the catalytic role of the activation loop may vary in mechanistic detail, but clearly all indicate the dominant role of the auto-inhibitory domain. Another possible explanation is that the observed mobility change may be associated with the monomeric form of the enzyme complex explored here, since the role of dimerization of KD-AID has not been completely ruled out in the experimental studies.

In this work, we have shown that the AID inhibits catalytic function by restraining KD to an inactive-open state, thereby limiting functional local structural rearrangements, and providing dynamical support for the conformational switch mechanism of auto-inhibition of AMPK. Although our dynamical study is based on the AMPK α-subunit homolog Snf1 in *S.Pombe*, and not the AMPK α-subunit itself, it is reasonable to apply this dynamical mechanism to mammalian AMPK since Chen and co-authors [12] have already shown that the structure used in our study, rat AMPK α-subunit and rat AMPK holoenzyme, have the same auto-inhibition mechanism through extensive homologous mutagenesis analysis. However, care should be taken when using this enzyme model system. There still are many uncertainties about the relevance of the *S.Pombe* KD-AID fragment to the mammalian system, such as the role of the unfolded αG which is obviously different from other AMPK homologs, and the unknown impact of phosphorylation of the activation loop on the resulting catalytic function. In addition, the auto-inhibition mechanism of AMPK in other species could be different from *S.Pombe* Snf1 and rat AMPK due to considerable AID sequence divergence. For example, a recent structure of *S.Cerevisiae* AMPK homolog Snf1, containing the KD, AID and a region mediating interactions with β and γ subunits, shows a new inhibited conformation with a completely disordered AID [18]. To summarize, the dynamical mechanism of auto-inhibition explored in our work needs further investigation in other systems to ensure its robustness and applicability for mammalian AMPK.

## Methods

### Normal mode structural similarity

In our previous paper [15], we reported a new protein structural similarity metric defined by normal modes calculated from an elastic network model from structures drawn from a molecular dynamics trajectory. Here we briefly review the model and method. The potential energy used to calculate normal mode is a pairwise Hookean potential proposed by Tirion [19]:
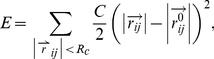
(1)where 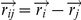, 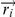 and 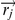 are 3 dimensional vectors for atom *i* and *j*, 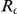 is an cutoff determining the number of elastic springs in total potential, and *C* is a phenomenological constant. The zero superscript indicates the reference structure. Then the second derivative of total potential energy with respect to atomic coordinates yields
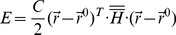
(2)where 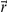 is the 3N-dimentional vector representing the Cartesian coordinates of N atoms, and *H* is the Hessian matrix, that when diagonalized, generates the normal mode eigenvectors, 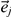, and corresponding eigenvalues (frequencies), 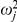.

In Tirion's potential model, the Hessian matrix *H* and corresponding normal modes and frequencies depend only on the given reference structure and cutoff, so the normal modes and corresponding frequencies can be calculated for each given conformation. For two different configurations 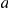 and 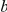 of a given molecule, the normal modes and frequencies of each conformation can be calculated from an elastic network model (ENM) under the assumption that every configuration is treated as a reference structure independently. We then fix the frequency order of all normal modes, 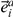, in set 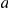
_,_ and then search for the normal mode 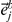 in set 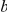
_,_ with the highest overlap to the low frequency mode 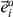, i.e.
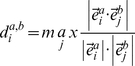
(3)where 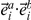 is the inner product between two normal modes. The structural similarity between conformations *a* and *b* can be defined as:
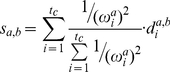
(4)where 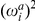 is the frequency of target normal mode 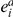, and 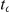 is number of chosen normal modes. The value of 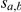 varies between 0 and 1, where larger values correspond to greater similarity than smaller values.

We use a relatively simple approach of taking the maximum in Eq. (3) to match the normal modes between two sets of configurations, which works very well if only a limited number of low frequency elastic normal modes are chosen to calculate the structural similarity, in agreement with another recent analysis of principle component analysis and normal mode analysis for capturing relevant protein dynamics [20]. In addition, the frequencies of the 2–3 normal modes are used as weights in Eq. (4), which we have found adds robustness for the stability of the INM similarity metric. We do this to solve the “mode shuffling” problem, although a more sophisticated method, the root mean-square inner product (RMSIP) with optimal mixing [21], could be used to better solve the mode matching problem and to reduce dependency on fine structural detail. In order to test sensitivity to structural detail, we calculated the frequencies of the reference open state (PDB Id: 3H4J) to the counterpart of energy-minimized one (see MD simulation subsection). We found that the frequencies differed by less than 1%, which suggests our approach is not strongly influenced by artifacts of fine structural details.

### Configurational entropy calculation

Schilitter introduced a simulation trajectory-based method to calculate the configurational entropy in the Cartesian coordinates by using the covariance matrix [22], in which the configurational entropy S_conf_ is approximated by
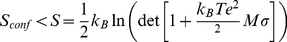
(5)where 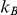 is Boltzmann's constant, T is the temperature, *e* is Euler's number, 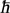 is Planck's constant divided by 2T, M is 3N-dimensional diagonal atomic mass matrix with each atomic mass occupying 3 consecutive positions, and 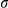 is the corresponding 3N-dimensional positional fluctuation covariance matrix. The elements of the covariance matrix, 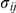, is
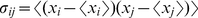
(6)where 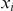 is the one dimensional Cartesian coordinate of the atom after the structural alignment.

### Molecular dynamics and INM simulations

Several different sequences and sub-structures for the open state were considered: wild-type KD-AID fragment (chain B of PDB structure: 3H4J), KD fragment (residues 27–297 of chain B of 3H4J), mutant 1 (N345A in 3H4J), mutant 2 (L341D in 3H4J), mutant 3 (M316E in 3H4J). Mutations were done by using the software package Molden [23].

All simulations were run with the Gromacs 4.0.3 package [24] using the OPLS-AA all atom force field [25]. Simulations were run with cubic periodic boundary conditions, Particle-Mesh Ewald [26] with 1 nm cutoff for the real-space electrostatic interactions, and a 1.2 nm cutoff for van der Waals interactions. The simulations were run in the NPT ensemble with a step size of 2 fs under the constant temperature (300 K) and pressure (1 bar) conditions by using the Berendsen coupling scheme [27] for both temperature and pressure. The LINCS algorithm [28] was used to constrain all bonds. We used anywhere between 20,000 to 25,000 TIP4P water [29] molecules, with the numbers of waters depending on the protein systems. An appropriate number of Na^+^ or Cl^−^ ions were added to neutralize the corresponding protein system. For the mutant systems, 50 steps of energy minimization were done with all heavy atoms being restrained by 10,000 kJ mol^−1^ nm^−1^ except mutated residue before adding any waters and ions to relax the residue mutated by software Molden [23] (KD-AID and KD fragment don't need this step). After addition of waters and ions, the steepest descent algorithm was run for 1,000 steps, followed by a 500 ps trajectory in which heavy atom positions were restrained by a strong harmonic force which is gradually relaxed from 1,000 kJ mol^−1^ nm^−1^ to zero.

Our sampling scheme involves generation of a relatively long 50 ns trajectory for each protein and its relevant start state (open or closed), which seed the initial state for ∼70–90 10 ns timescale MD simulations (run in parallel) that differ only in their regenerated velocities drawn from the Maxwell distribution. Instantaneous normal mode (INM) similarity is used to monitor all trajectories to observe any conformational changes to two reference end states 3H4J and 3DAE. We use a 13 Å cutoff in the evaluation of Eq. (1), and use the first two lowest frequency normal modes (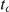 in Eq. (4)) evaluated against the 3H4J and 3DAE reference templates to calculate INM similarities for snapshots generated every 10 ps in all trajectories.

Residues 27–175 in chain B of PDB structure 3H4J is used as open reference structure and corresponding residues 48–196 in chain A of 3DAE (aligned by ClustalW [30]) is used as closed reference structure to measure the interlobe conformational transition, consistent with the experimental structural analysis [12]. Due to the missing residues 93–96 in PDB structure 3DAE, corresponding residues 72–75 in PDB structure 3H4J are also eliminated in the INM similarity calculations. Furthermore, the activation loop in PDB structure 3DAE is invisible, and residues 229–250 in 3H4J are coil while the corresponding residues 250–271 in 3DAE form a α-helix. Therefore we eliminated these two long coils in the evaluation of our INM similarity metric, to better capture more meaningful conformational changes. To confirm, we also did structural similarity calculations on the whole KD fragment by excluding these two long coils, and there is no significant difference from our chosen segment, residues 27–175 in 3H4J, (data not shown).

## Supporting Information

Text S1 **Supplemental figures and tables.** Figure 1. Backbone dihedral angle change for KD fragment. The hinge residue GLY115, connecting two lobes, transitions to active state even in global open conformation. Figure 2. Backbone dihedral angle change for mutant 2. The hinge residue GLY115, connecting two lobes, transitions to active state even in global open conformation. Figure 3. Backbone dihedral angle change for mutant 3. The hinge residue GLY115, connecting two lobes, transitions to active state even in global open conformation. Table 1. Backbone dihedral angle changes of functional residues in closed state, by comparing to open state. F denotes more flexible backbone in closed state than open state, T represents the occurrence of backbone structural transition during the global interlobe conformational transition from open to closed state, and C means that the backbone transitions to closed-active or nearly closed-active state, or prefers closed-active state if there are several conformational basins, by comparing to reference closed-active structure 3DAE. Y is for Yes and the blank means No.
(DOC)
