# Supplementary material for: The Dynamical Mechanism of Auto-Inhibition of AMP-Activated Protein Kinase
Source: PLoS Comput Biol. 2011 Jul 21;7(7):e1002082. doi: 10.1371/journal.pcbi.1002082 (PMC3140967; doi:10.1371/journal.pcbi.1002082)

Supplementary Tables and Figures

Supplementary Table 1. Backbone dihedral angle changes of functional residues in closed state, by comparing to open state. F denotes more flexible backbone in closed state than open state, T represents the occurrence of backbone structural transition during the global interlobe conformational transition from open to closed state, and C means that the backbone transitions to closed-active or nearly closed-active state, or prefers closed-active state if there are several conformational basins, by comparing to reference closed-active structure 3DAE. Y is for Yes and the blank means No.

|  |  | KD | | | Mutant 2 | | | Mutant 3 | | |
| --- | --- | --- | --- | --- | --- | --- | --- | --- | --- | --- |
|  | Res No. | F | T | C | F | T | C | F | T | C |
| Gly-rich loop | GLY41 |  | Y | Y |  |  |  |  |  |  |
|  | GLU42 | Y | Y | Y |  |  |  |  |  |  |
|  | GLY43 | Y | Y | Y |  |  |  |  |  |  |
|  | PHE45 | Y | Y | Y |  |  |  |  |  |  |
|  | LYS47 | Y | Y |  |  |  |  |  |  |  |
| Turn in sheets | HIS53 |  |  |  | Y | Y |  | Y |  |  |
|  | LYS55 |  |  |  |  |  |  | Y |  |  |
| Helix aC | ARG78 |  |  |  |  |  |  | Y |  |  |
|  | VAL79 |  |  |  |  |  |  | Y | Y |  |
|  | GLU80 | Y |  |  |  |  |  |  |  |  |
|  | ARG81 |  | Y |  |  |  |  |  | Y |  |
|  | GLU82 |  |  |  |  |  |  |  | Y |  |
| Turn after aC | HIS91 |  |  |  |  | Y |  |  |  |  |
| Hinge of two lobes | GLY115 |  |  | Y |  |  | Y |  |  | Y |
|  | GLY116 | Y | Y |  |  |  |  | Y |  | Y |
|  | GLU117 |  |  |  | Y |  | Y |  |  |  |
| Catalytic loop | VAL153 |  | Y | Y | Y | Y | Y |  |  |  |
|  | HIS154 | Y |  |  | Y | Y | Y |  |  |  |
|  | ARG155 | Y | Y |  | Y | Y |  |  |  |  |
|  | ASP156 | Y | Y | Y | Y | Y |  |  |  |  |
|  | LEU157 |  | Y | Y | Y | Y |  |  |  |  |
|  | LYS158 |  | Y | Y |  | Y |  |  |  |  |
| DFG-motif | ASP174 | Y | Y |  |  |  |  |  | Y |  |
|  | PHE175 | Y | Y |  |  |  |  | Y | Y |  |
|  | GLY176 |  |  |  | Y |  |  |  | Y |  |

supplementary figure 1. Backbone dihedral angle change for KD fragment. The hinge residue GLY115, connecting two lobes, transitions to active state even in global open conformation.


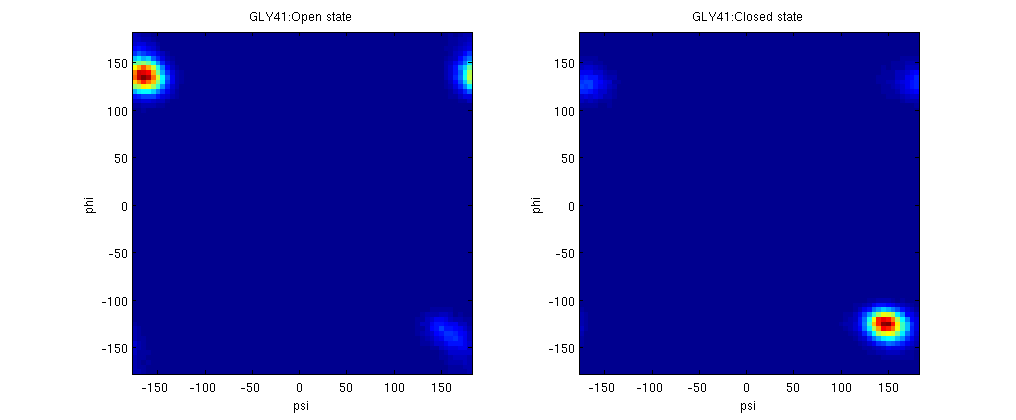


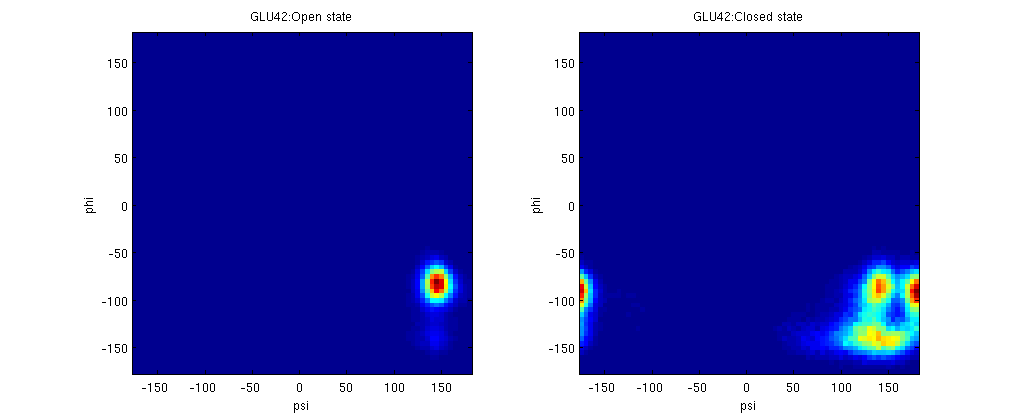


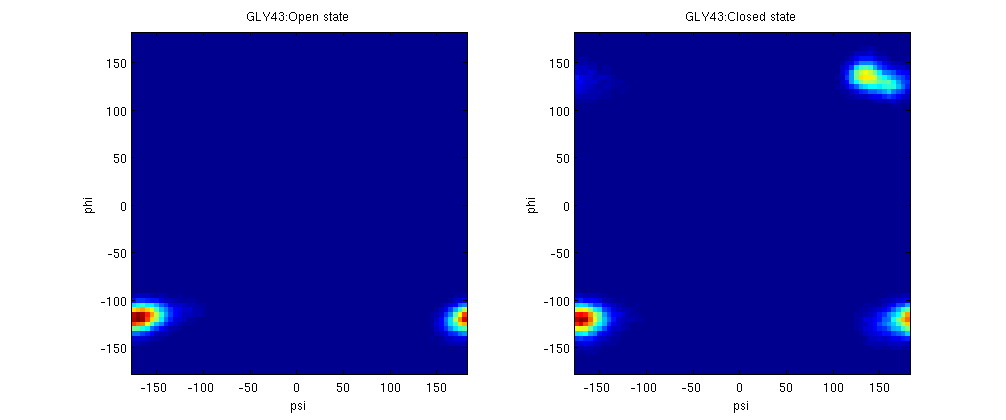


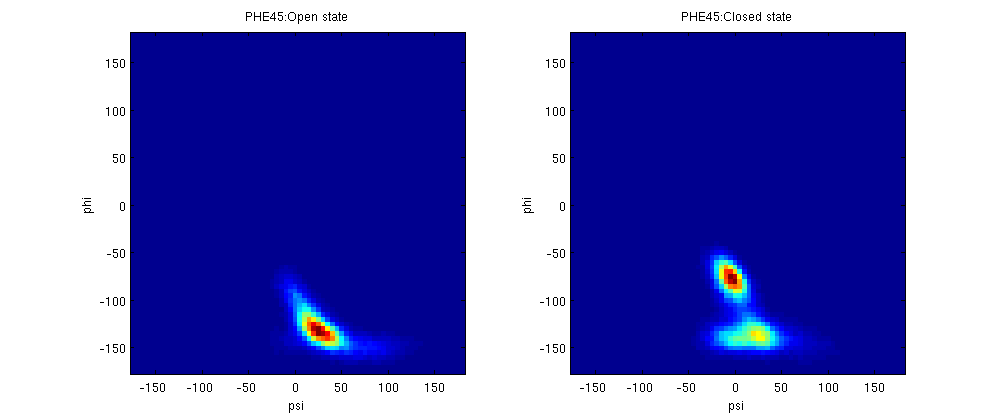


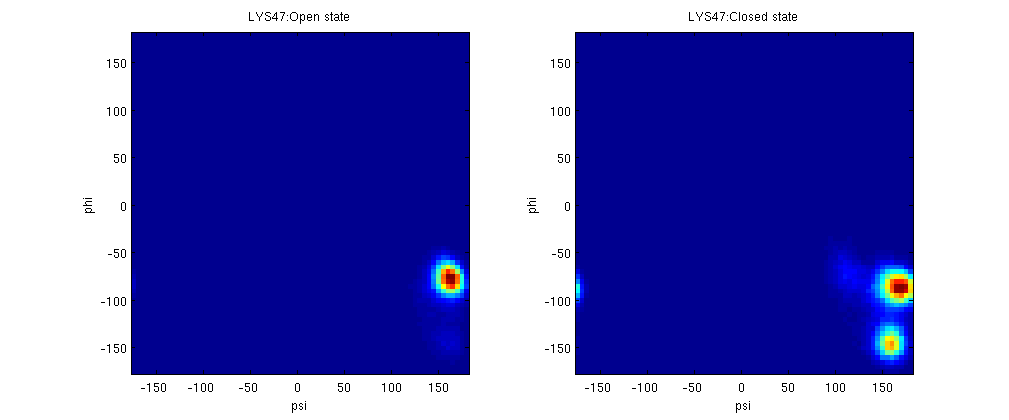


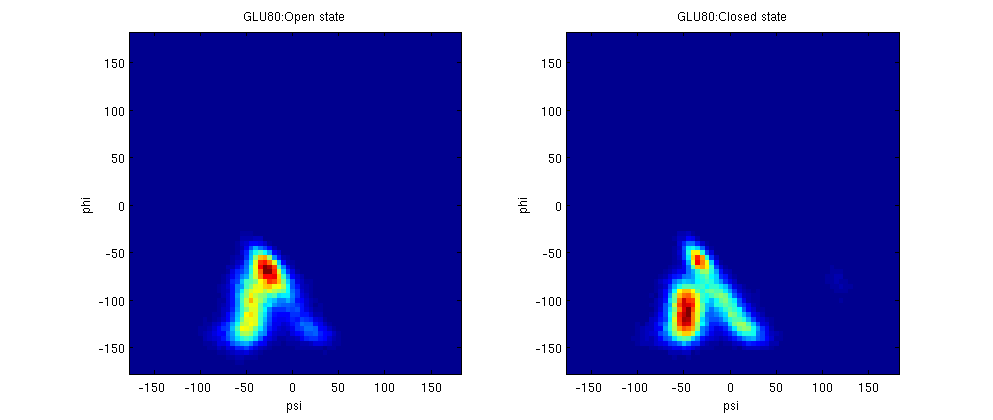


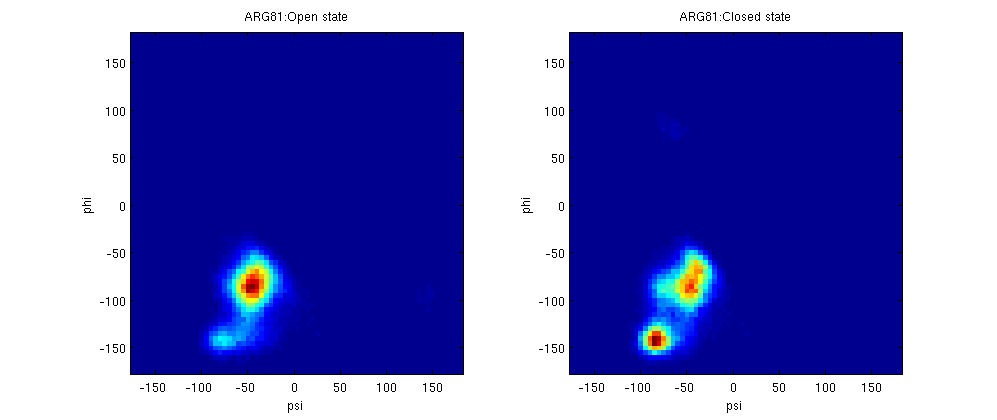


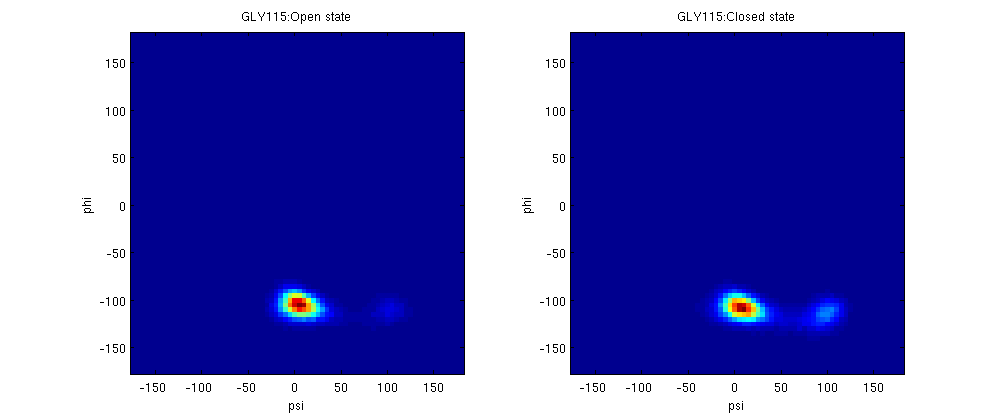

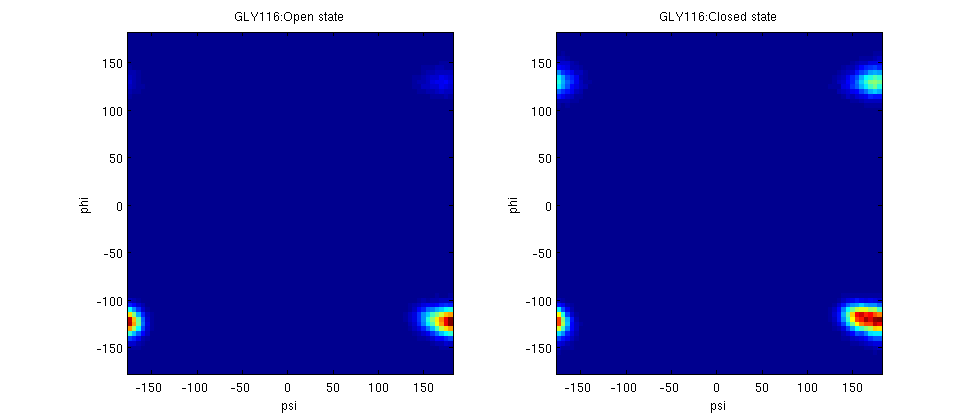

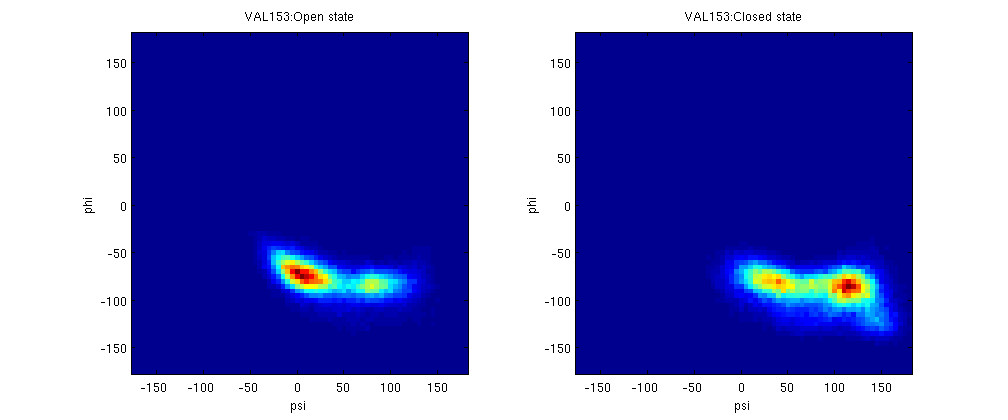

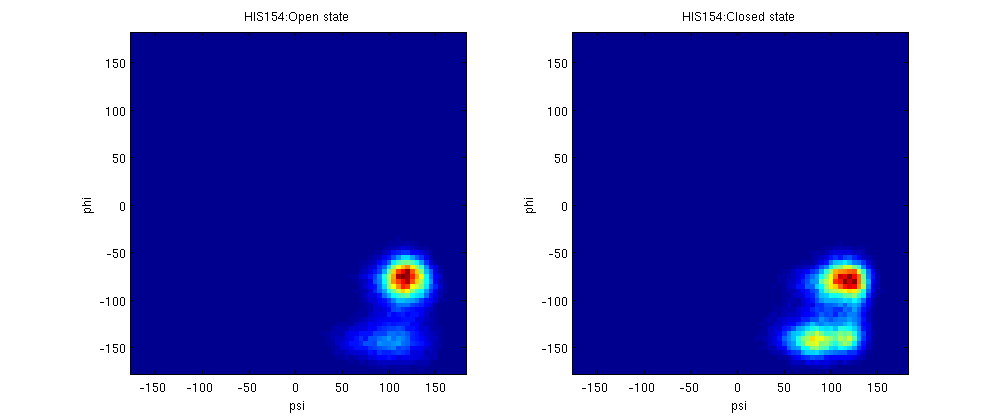


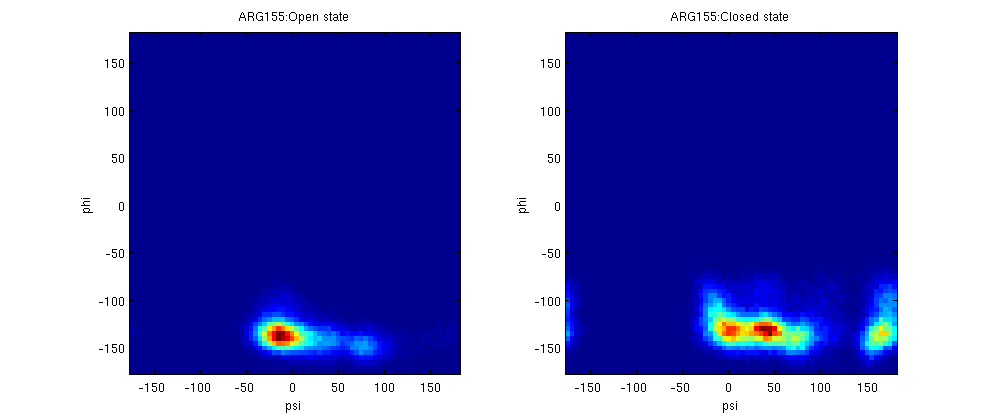

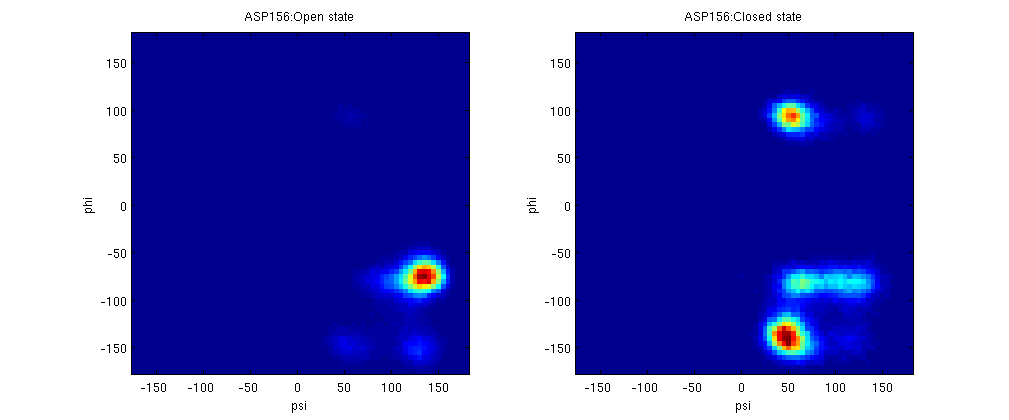

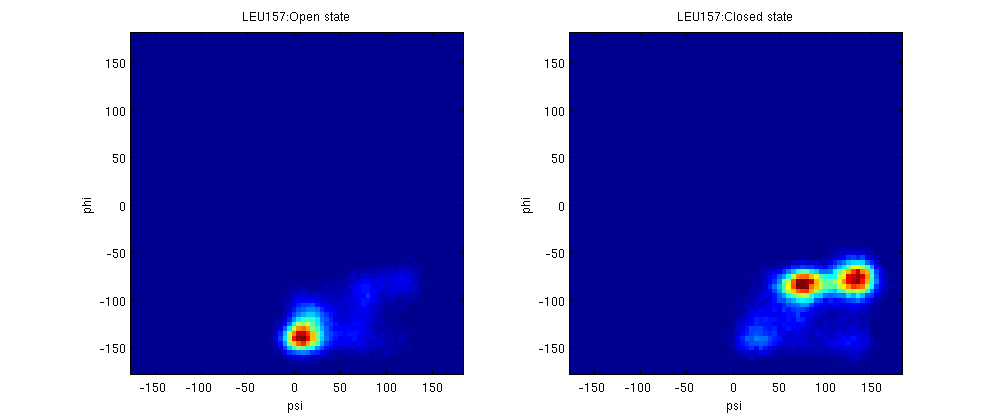

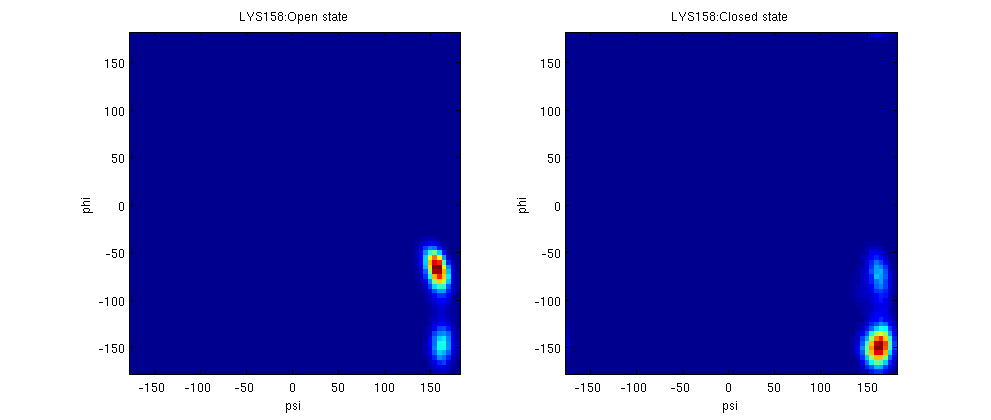


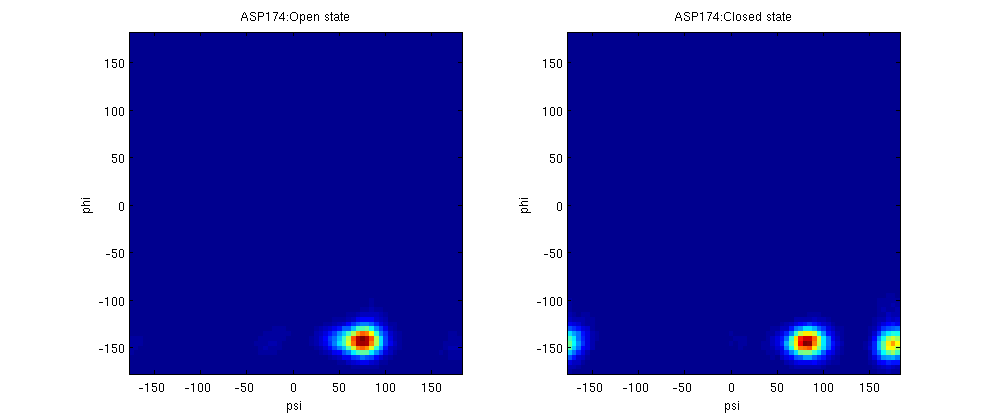

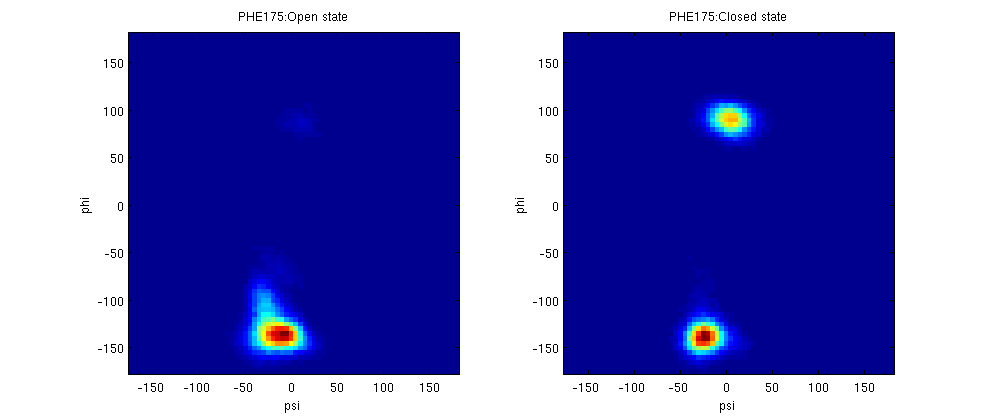


supplementary figure 2. Backbone dihedral angle change for mutant 2. The hinge residue GLY115, connecting two lobes, transitions to active state even in global open conformation.


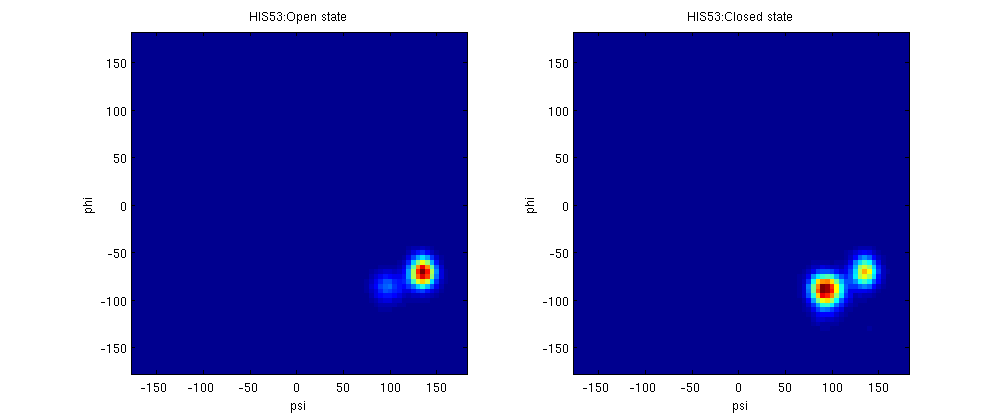

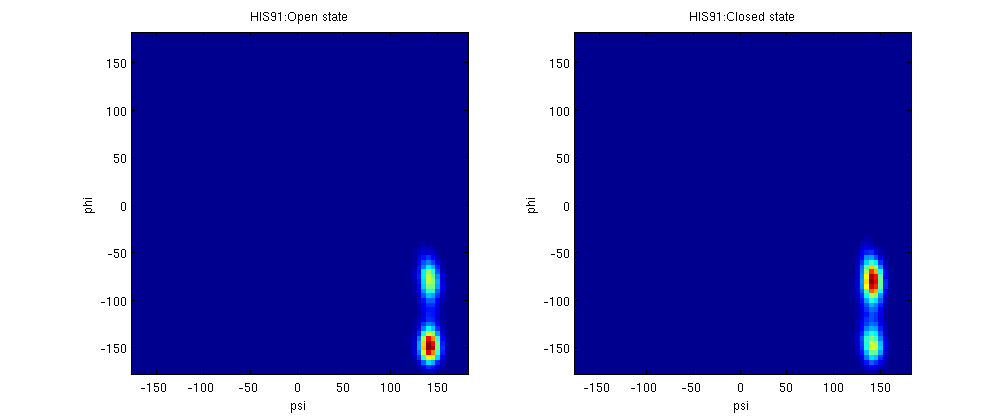

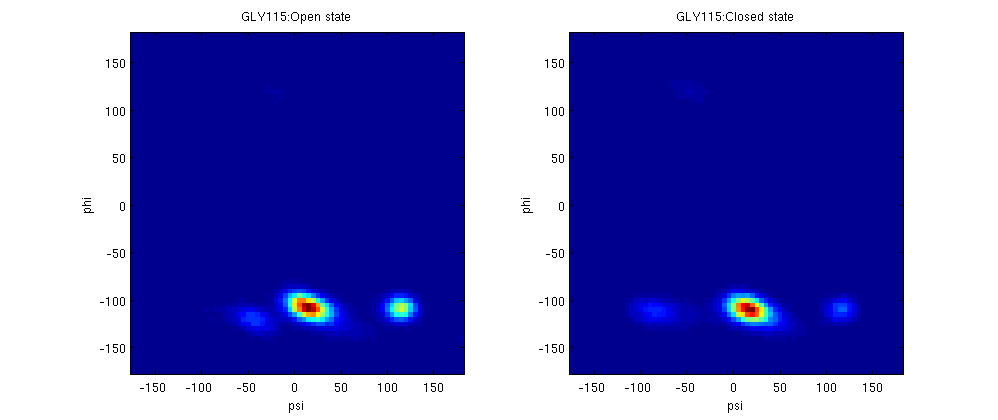


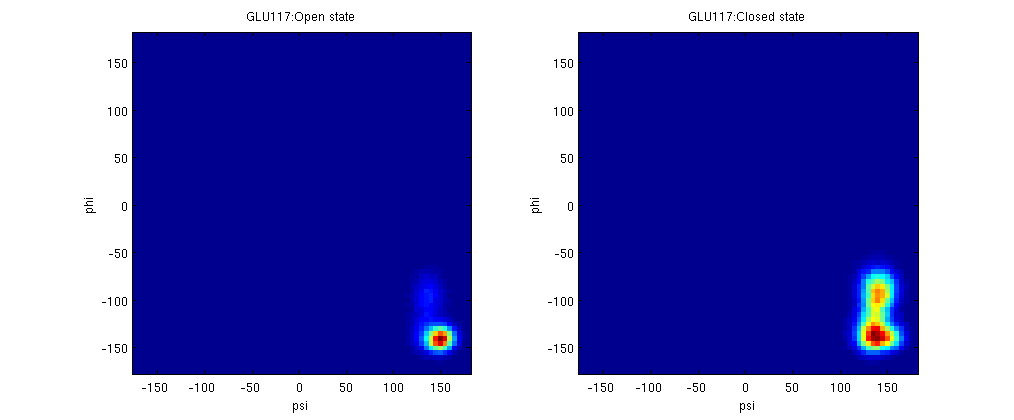


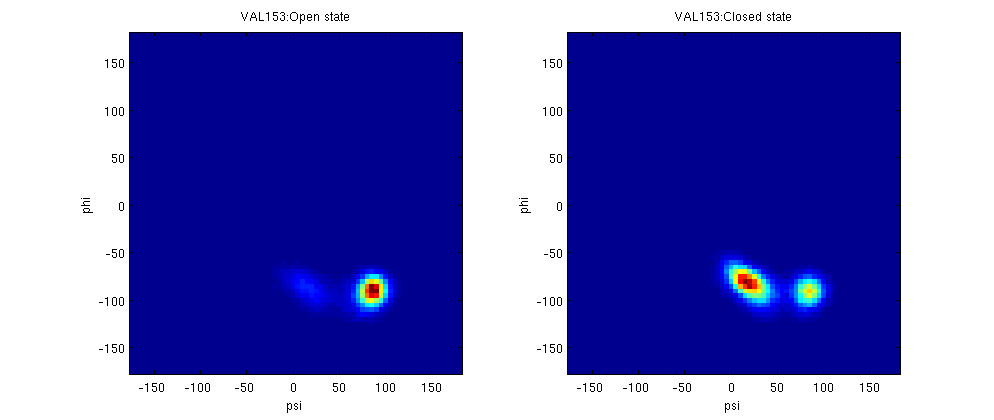

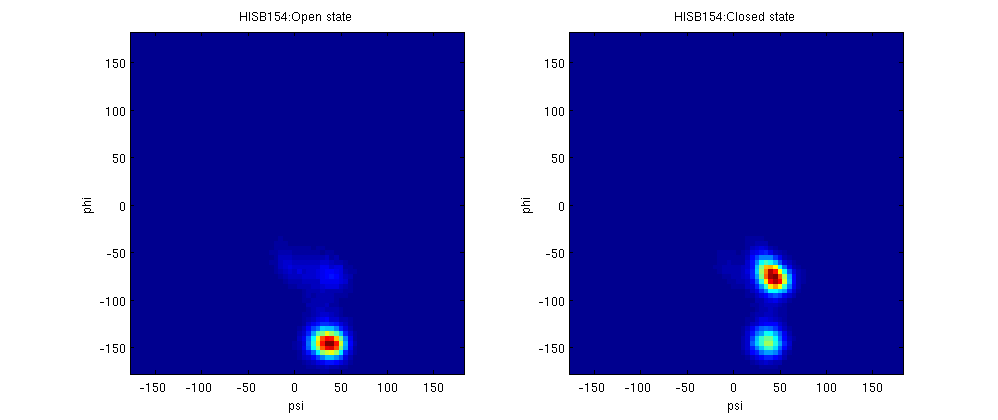

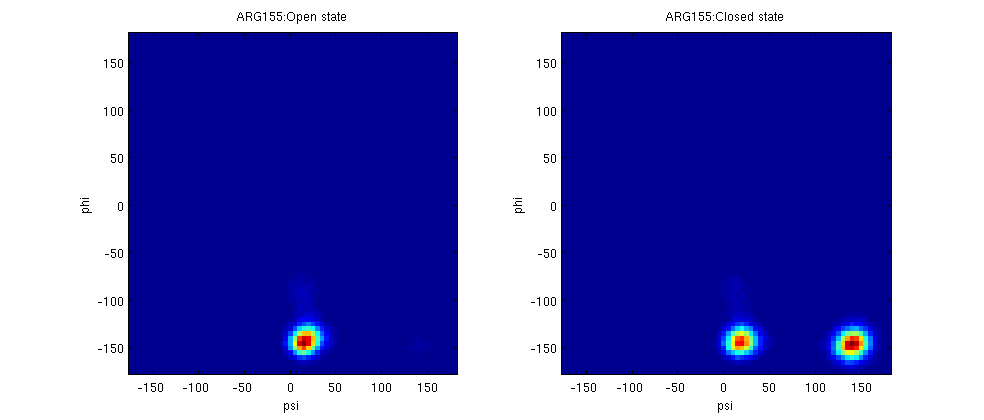


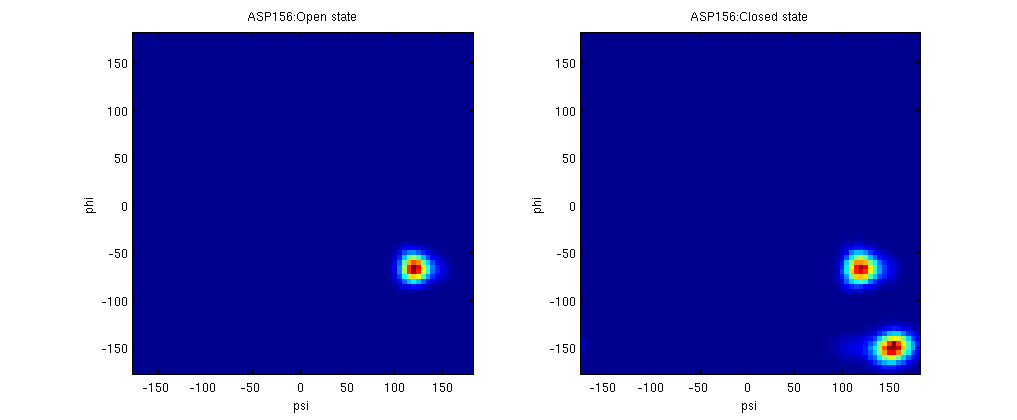

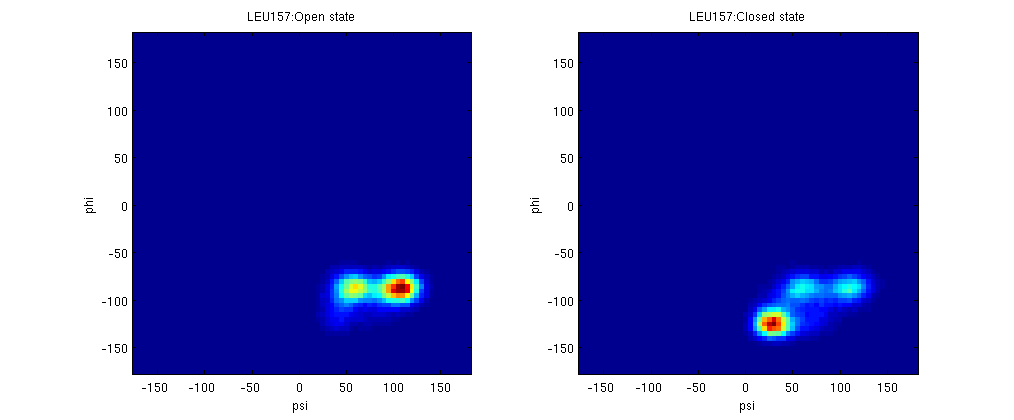

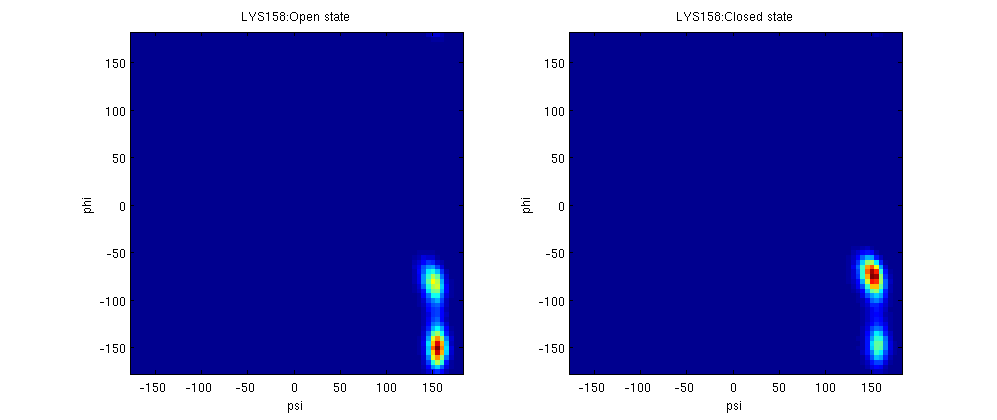

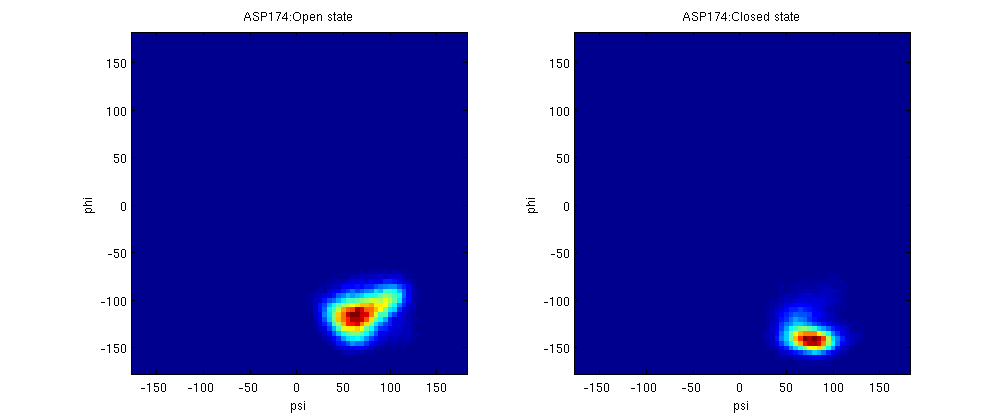

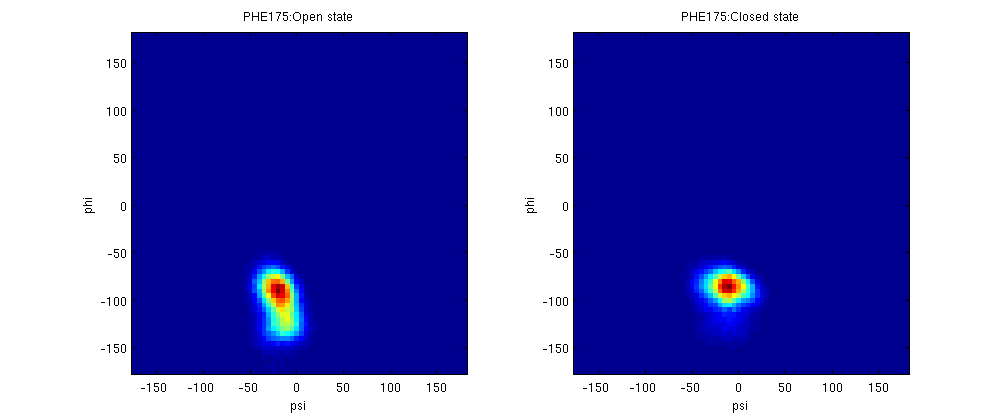


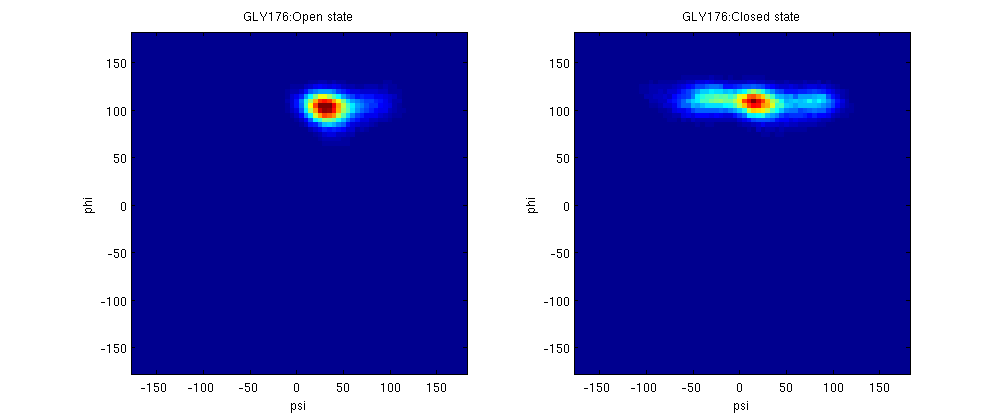


supplementary figure 3. Backbone dihedral angle change for mutant 3. The hinge residue GLY115, connecting two lobes, transitions to active state even in global open conformation.


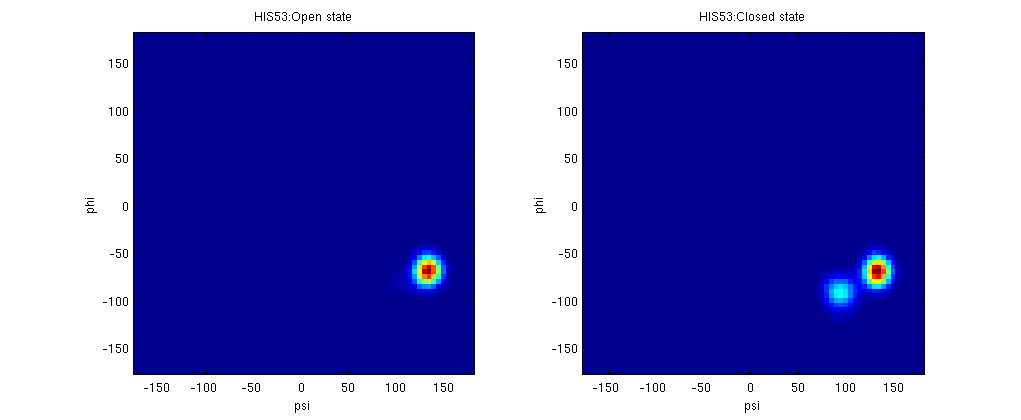

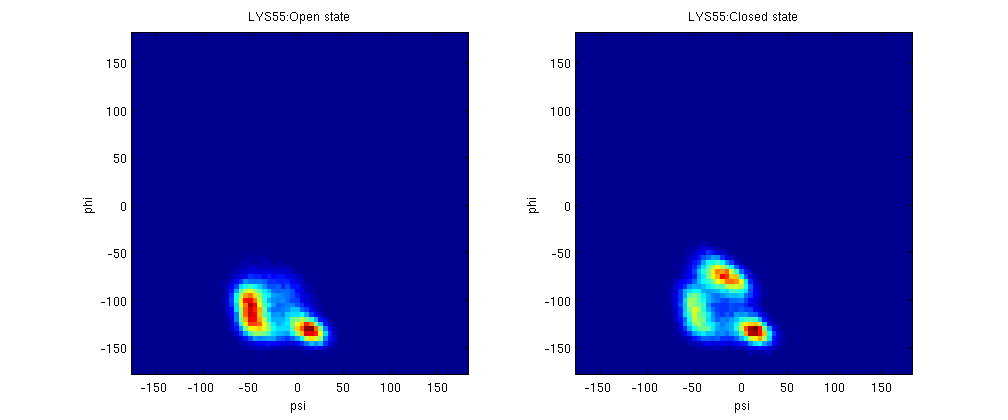

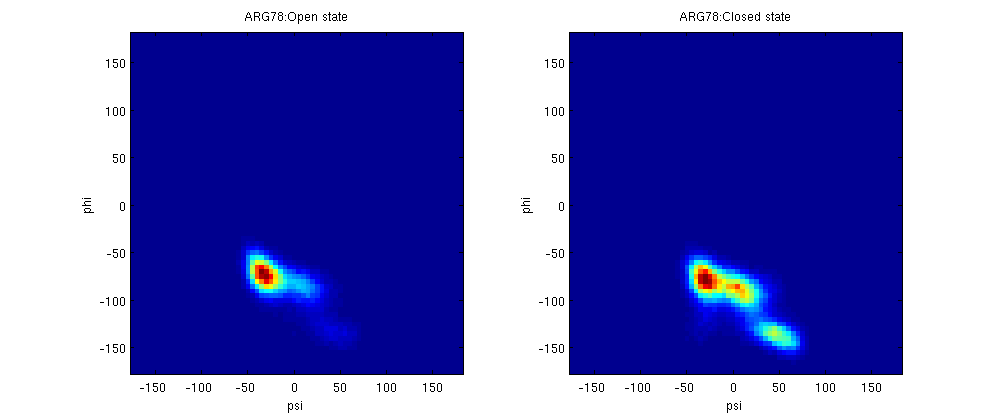


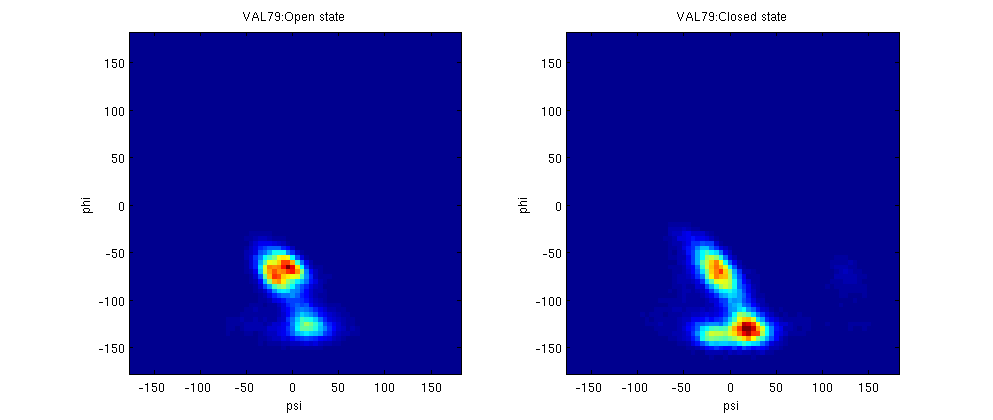

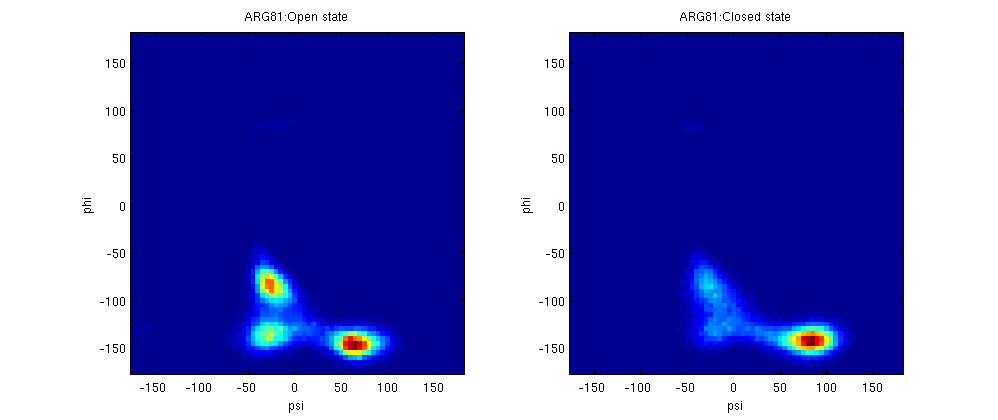

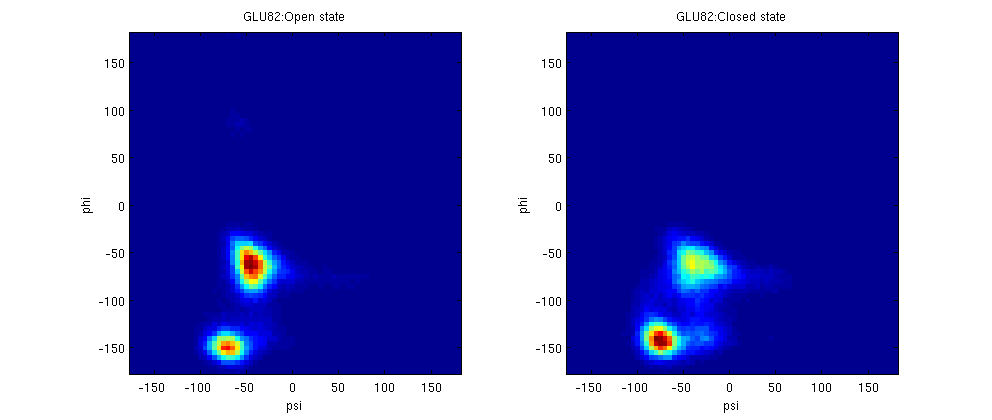

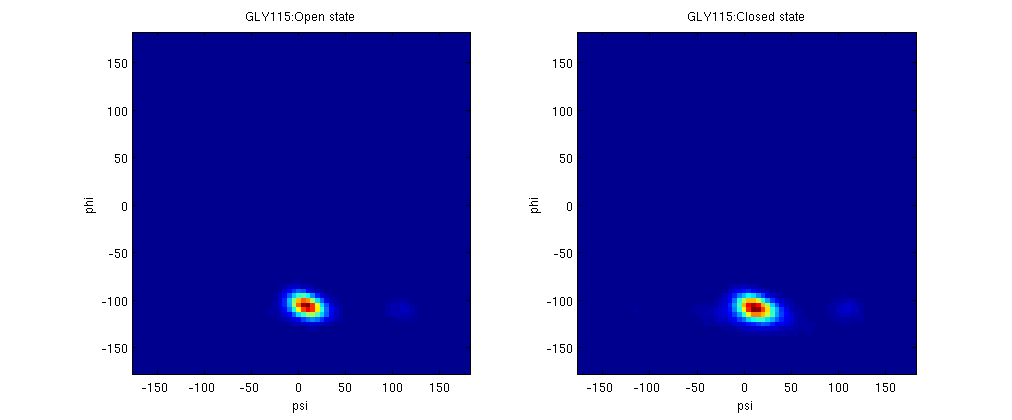


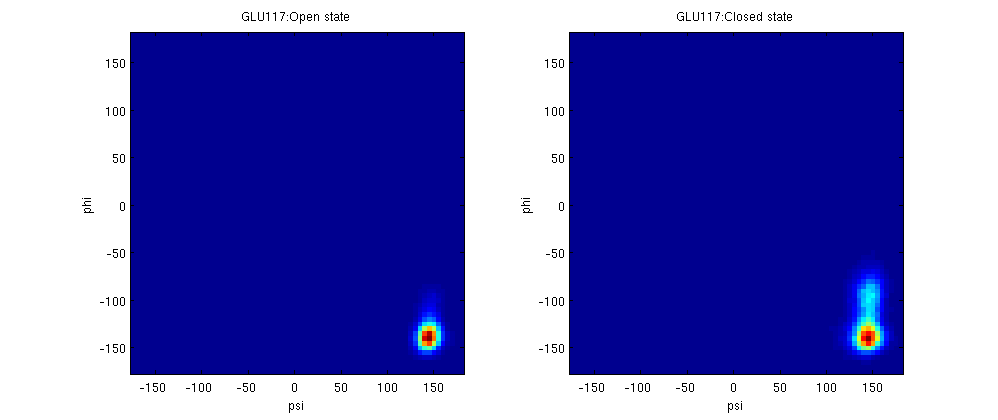

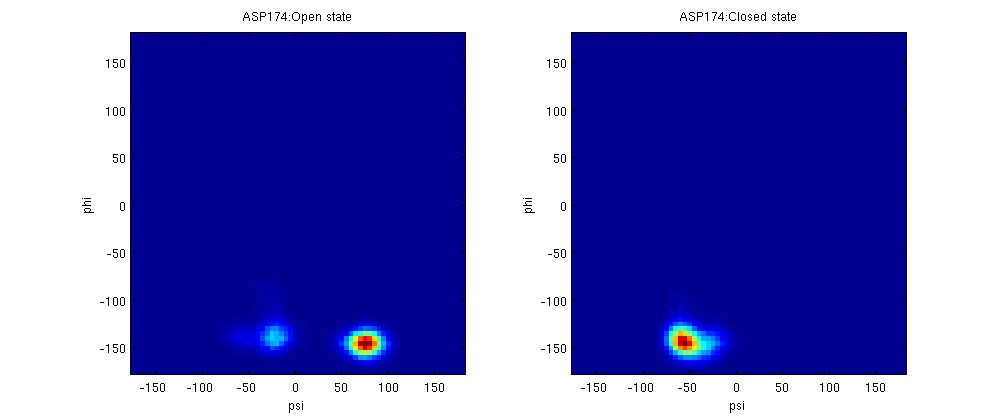

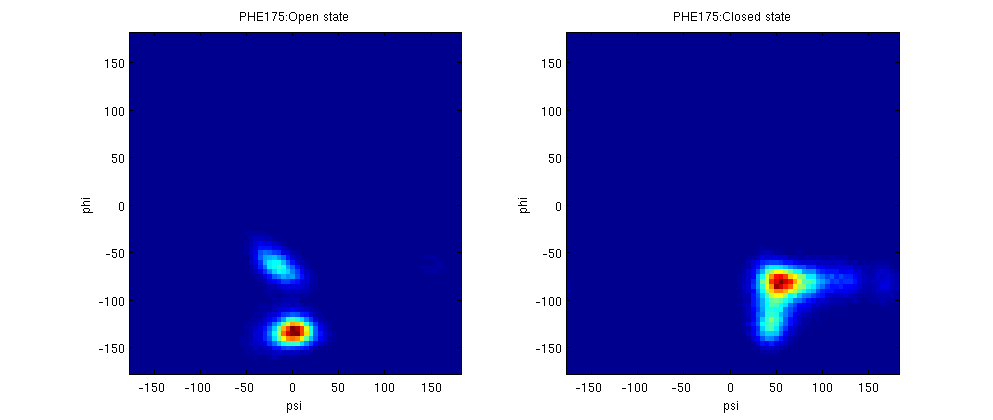

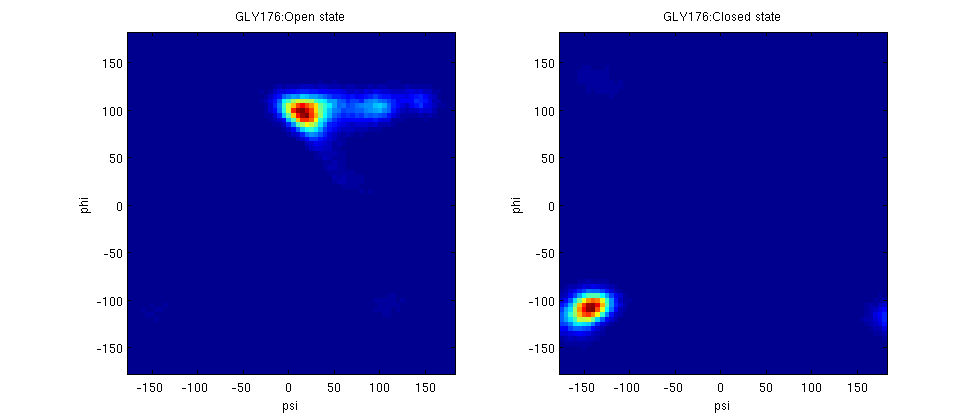

Supplement: Text S1 — Supplemental figures and tables. Figure 1. Backbone dihedral angle change for KD fragment. The hinge residue GLY115, connecting two lobes, transitions to active state even in global open conformation. Figure 2. Backbone dihedral angle change for mutant 2. The hinge residue GLY115, connecting two lobes, transitions to active state even in global open conformation. Figure 3. Backbone dihedral angle change for mutant 3. The hinge residue GLY115, connecting two lobes, transitions to active state even in global open conformation. Table 1. Backbone dihedral angle changes of functional residues in closed state, by comparing to open state. F denotes more flexible backbone in closed state than open state, T represents the occurrence of backbone structural transition during the global interlobe conformational transition from open to closed state, and C means that the backbone transitions to closed-active or nearly closed-active state, or prefers closed-active state if there are several conformational basins, by comparing to reference closed-active structure 3DAE. Y is for Yes and the blank means No. (DOC) [file pcbi.1002082.s001.doc]
